# Disparities in male versus female oncologic outcomes following bladder preservation: A population‐based cohort study

**DOI:** 10.1002/cam4.3835

**Published:** 2021-03-28

**Authors:** Leslie K. Ballas, Stephanie Navarro, Chunqiao Luo, Croix C. Fossum, Albert Farias, Siamak Daneshmand, Susan Groshen

**Affiliations:** ^1^ Department of Radiation Oncology Keck School of Medicine University of Southern California Los Angeles CA USA; ^2^ Department of Preventative Medicine Keck School of Medicine University of Southern CA Los Angeles CA USA; ^3^ Department of Urology Keck School of Medicine University of Southern California Los Angeles CA USA

**Keywords:** bladder preservation, muscle‐invasive bladder cancer, sex‐based disparities, trimodality therapy

## Abstract

**Introduction:**

In surgical series of muscle‐invasive bladder cancer (MIBC), women have higher recurrence rates, disease progression, and mortality following radical cystectomy than men. Similar reports of oncologic differences between men and women following trimodality therapy (TMT) are rare. Our hypothesis was that there would be no difference in overall survival (OS) between sexes receiving TMT.

**Methods:**

We queried the National Cancer Database (NCDB) for patients diagnosed with clinical stage T2‐T4aN0 M0 MIBC between 2004–2016. We considered patients to have received TMT if they received 55 Gy in 20 fractions or 59.4–70.2 Gy of radiotherapy with concurrent chemotherapy following a transurethral resection of bladder tumor (TURBT). We used multivariable Cox proportional hazard models to determine whether sex was associated with risk of mortality. In addition to OS, we calculated relative survival (RS) to adjust for the fact that females generally survive longer than males.

**Results:**

Of the patients, 1960 underwent TMT and had survival data. Less than one quarter were female. In the first year following treatment, women had worse OS and RS than men (*p* = 0.093 and *p* = 0.030, respectively). However, overall and relative survival differences between sexes were not statistically significantly different in Years 2 and later. Unlike with OS, the RS between sexes remained significant at 9 years; in multivariable analysis based on RS, women were 43% more likely to die than men (*p* < 0.001).

**Conclusions:**

Women had a higher initial risk of death than men in the first year following TMT. However, long‐term survival between sexes was similar. TMT is an important treatment option in both men and women seeking bladder preservation.

## Graphical abstract

In surgical series of muscle invasive bladder cancer, females have higher recurrence rates, disease progression, and mortality following radical cystectomy than men. Trimodality therapy (TMT) is an important definitive treatment option in patients seeking bladder preservation. This study examines survival following TMT of women compared with men in a population‐based analysis.

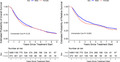

## 1 INTRODUCTION

Bladder cancer is the fourth most common cancer in men and will lead to an estimated 17,980 deaths in the United States in 2020. The incidence of bladder cancer is about four times higher in men than in women.^1^ Although there is a higher incidence in men, studies show that women have more advanced disease at the time of diagnosis.^2^ In the time leading up to definitive bladder cancer treatment, there are reported disparities between men and women in the promptness and thoroughness of initial diagnostic evaluation.^3^ These sex‐based disparities create delays for women in the workup of hematuria,^4^ the timeliness of abdominopelvic imaging,^4^ and referrals to urology.^5^ Delays in diagnosis can lead to increased cancer‐specific mortality among patients with a longer time from initial hematuria to bladder cancer diagnosis.^6^ Additionally, the distribution of molecular subtypes is different between the sexes^7^ and may lead to differential oncologic outcomes.

Surgical series with thousands of patients have reported that females with muscle‐invasive bladder cancer (MIBC) have higher recurrence rates, disease progression, and mortality following radical cystectomy (RC) compared to men.^8^, ^9^ However, to our knowledge, there are no data examining whether these sex disparities exist in patients who undergo trimodality therapy (TMT). A pooled analysis of Radiation Therapy Oncology Group (RTOG) TMT studies did not demonstrate a difference in disease‐specific survival (DSS) or overall survival (OS).^10^ Moreover, BC2001, a randomized trial comparing TMT to RT alone, did not report differences in outcomes based on sex.^11^ Outside of clinic trials, differences in outcomes from TMT between the sexes in clinical practice are largely unknown. To address the question of sex‐based disparities in patients with MIBC who received TMT, we compared outcomes of between male and female cohorts using the National Cancer Database (NCDB). Our hypothesis was that a population‐based analysis would reflect what has been observed in clinical trials and there would be no difference in OS between men and women receiving TMT for MIBC.

## 2 METHODS

### 2.1 Data source

The NCDB is a hospital‐based registry maintained by the American Cancer Society and the American College of Surgeons and captures about 70% of all patients who are newly diagnosed with cancer.^12^, ^13^ The NCDB includes deidentified data including demographics, comorbidities, tumor characteristics, therapies delivered (including surgery, radiotherapy, chemotherapy, and multimodal treatment), and survival data.^14^, ^15^

### 2.2 Study design and patient selection

**FIGURE 1:**
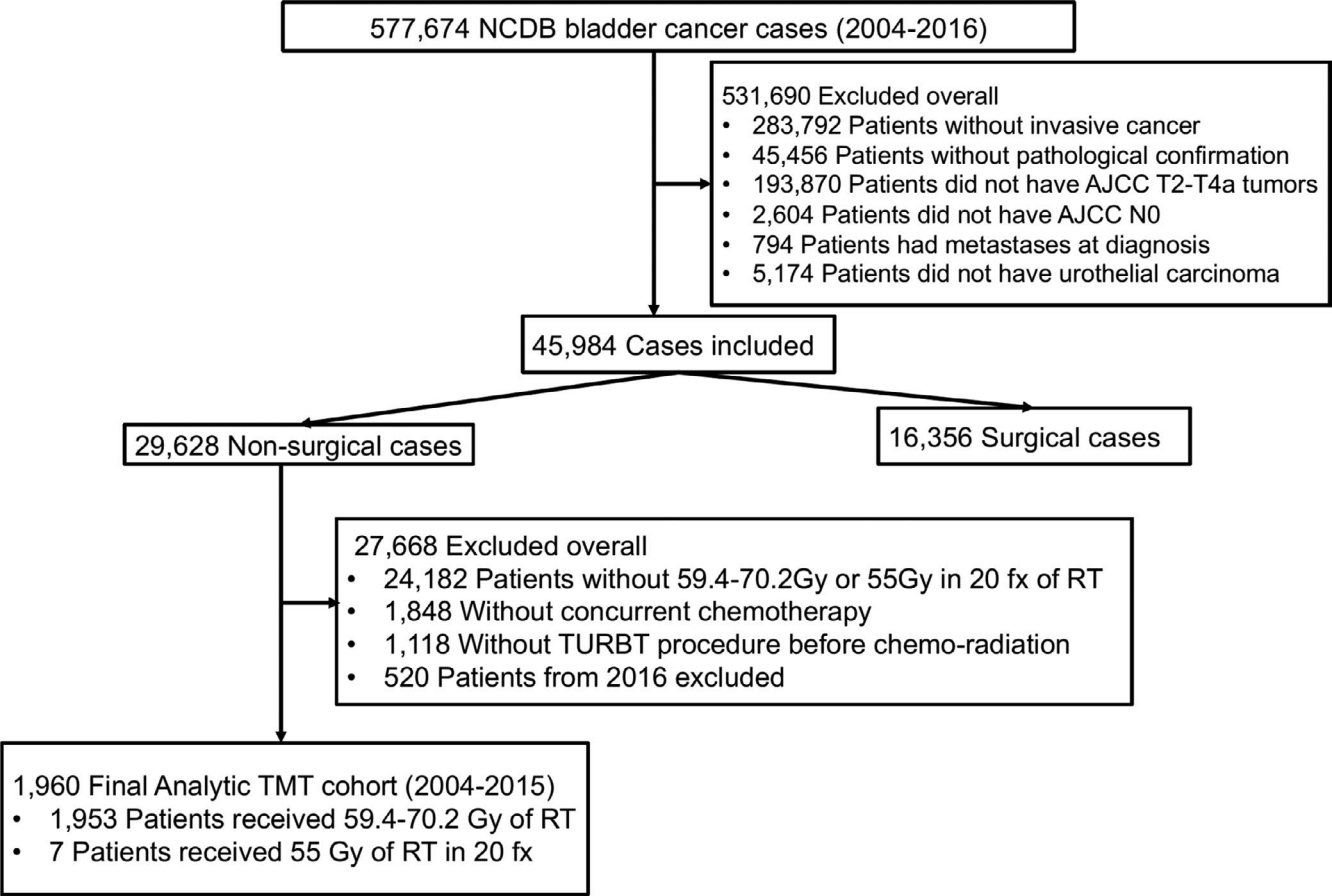
Flow chart of identification of patients with confirmed AJCC cT2‐T4aN0 M0 urothelial cancer of the bladder who underwent TMT during the years of 2004 and 2016

This was a retrospective cohort study of the subset of patients in the NCDB who underwent TMT as their definitive treatment for newly diagnosed MIBC. Using NCDB data files from 2004 to 2016, we queried the participant user file (PUF) for all patients with pathologically confirmed cT2‐T4a, N0, M0 urothelial cell carcinoma of the bladder. TMT was defined as radiotherapy to a dose of 55 Gy in 20 fractions or 59.4–70.2 Gy (current curative radiotherapy doses) with concurrent chemotherapy following maximal transurethral resection of bladder tumor (TURBT). Concurrent chemotherapy was defined as chemotherapy delivered during the first course of treatment along with radiotherapy delivered during the first course of treatment. The final analytic data included patients from 2004 to 2015 only. Patients who were diagnosed at 2016 did not have vital status available. Figure 1 depicts inclusion and exclusion criteria used to define our study cohort.

The dependent variables are OS and relative survival (RS). OS was calculated as the number of years from the date of start of TMT to the date of death of any cause, or date that the patient was last known to be alive, if the patient was not known to have died. The date of start of treatment was not known for 18 patients, so the date of diagnosis for these 18 patients was used as substitute. RS calculation methods are described in detail in statistical analysis.

The main independent variable of interest was sex, which was defined as male or female from the NCDB, and is a self‐reported measure abstracted from hospital encounter data. Covariates included age at diagnosis of MIBC, race, household income, treatment facility location, residential setting, Charlson/Deyo score, insurance type, treatment facility type, and distance between zip code and treating facility (miles). American Joint Committee on Cancer (AJCC) 7th Edition clinical T‐staging was also considered as a covariate.

### 2.3 Statistical analysis

Descriptive statistics were reported for both males and females who received TMT. Chi‐squared tests were used to assess sex differences for categorical variables. Wilcoxon rank sum tests were used to assess sex differences for continuous variables.

OS for males and females was estimated and displayed using Kaplan–Meier product limit calculations. Because it is known that in general, females survive longer than males, the possibility existed that the observed similar overall long‐term survival reflected a decrease in the higher expected survival of females. To evaluate this, the RS was calculated for males and females. RS probability was calculated as the ratio of the observed survival probability of the TMT‐treated patients compared to the expected survival probability based on the general population derived from life tables published by the Centers for Disease Control and Prevention (CDC) in 2004–2015, matched for gender, race (White, Black, and other), and the age of the patient and year TMT was started. Since CDC did not provide separate life tables for racial minorities except for Blacks during the years of 2004–2006, all other ethnic and racial minorities were matched using the total population life tables. All RS analyses were done by the R package “relsurv”; method “ederer2” was selected to estimate RS probabilities over time in males and females.^16^, ^17^

Because patterns in the survival and hazard plots suggested a lack of proportional hazards (i.e., a difference between males and females that changed over time), which could blunt an early difference between males and females, conditional probabilities of surviving were calculated. Computationally, conditional probabilities of surviving beyond a time *t* were based on the subset of patients who had survived up until time *t*, with survival recalculated as beginning at time *t*. Conditional patterns were summarized using the same methods used for unconditional analyses; comparison of males and females were based on the logrank test for conditional OS and a logrank‐like test for conditional RS.^17^

Andersen et al.^16^ univariable and multivariable Cox regression was implemented to determine risk factors for RS, while the standard univariable and multivariable Cox regression was implemented to determine risk factors for OS. For the multivariable methods, 98 (3.8%) patients (74 males and 24 females) were excluded because of a missing value for at least one covariate. R version 3.6.0 was used to conduct all analyses (R Foundation for Statistical Computing, Vienna, Austria). All *p* values are based on two‐sided tests.

## 3 RESULTS

A total of 45,984 MIBC cases were identified for potential inclusion. Among these, 1960 patients underwent radiotherapy to a dose of 55 Gy in 20 fractions or 59.4–70.2 Gy with concurrent chemotherapy after completing a TURBT (Figure 1). Slightly less than one quarter of patients were female (1488 males and 472 females), and this was consistent across treatments received. Of the patients who received TMT, survival/follow‐up information was missing on 520 patients; these patients are excluded from all analyses.

### 3.1 Patient characteristics

Characteristics of the 1960 patients undergoing TMT with survival/follow‐up information are shown in Table 1. Women tended to be slightly older: the median age was 77 years for males and 78 years for females (*p* = 0.030). Both male and female patients were largely White (93% of males, 86% of females), with a greater proportion of Black females than Black males (12% vs. 4.2%). Males were slightly more likely to have at least one comorbid condition (36% vs. 31%) compared to females. The majority of patients receiving TMT were diagnosed with cT2 bladder cancer (83% of males and females). Both men and women were most likely to receive TMT at comprehensive community cancer centers. Men lived farther away from treating facility than women, with median distance of 8 versus 6 miles, *p* < 0.001. The median dose of RT was the same for women and men (64.8 Gy), yet women were more likely to get a lower median dose of RT within the specified dose range included in this study (*p* = 0.037).

**TABLE 1:** Characteristics of patients receiving trimodality therapy group. *N* (%) or median (Q1, Q3)

Total number	Male *N* = 1488	Female *N* = 472	*p*
Age at diagnosis in years	77 (69, 83)	78 (70, 83)	0.030
Race^a^			<0.001
White	1386 (93%)	407 (86%)	
Black	63 (4.2%)	56 (12%)	
Asian/other/unknown	39 (2.6%)	9 (1.9%)	
Total RT dose	6480 (6300, 6480)	6480 (6166, 6480)	0.037
Income			0.72
Less than $38,000	237 (16%)	82 (17%)	
$38,000–$47,999	346 (23%)	117 (25%)	
$48,000–$62,999	400 (27%)	118 (25%)	
$63,000+	494 (33%)	153 (33%)	
Number of missing	11	2	
Urban/rural			0.40
Metro	1204 (83%)	387 (84%)	
Urban	218 (15%)	65 (14%)	
Rural	33 (2.3%)	6 (1.3%)	
Number of missing	33	14	
Charlson/Deyo score			0.091
No comorbid conditions recorded	949 (64%)	324 (69%)	
1	371 (25%)	95 (20%)	
Greater than or equal to 2	168 (11%)	53 (11%)	
Type of Insurance			0.45
Private insurance/managed care/other government	296 (20%)	85 (18%)	
Not insured/Medicaid	59 (4.0%)	15 (3.2%)	
Medicare	1120 (76%)	368 (79%)	
Number of missing	13	4	
Treatment facility type			0.89
Community cancer program	172 (12%)	50 (11%)	
Comprehensive community cancer program	693 (47%)	227 (48%)	
Academic/research program	410 (28%)	130 (28%)	
Integrated network cancer program	213 (14%)	64 (14%)	
Number of missing	0	1	
Treatment facility location			0.31
New England	99 (6.7%)	33 (7.0%)	
Middle Atlantic	264 (18%)	81 (17%)	
South Atlantic	316 (21%)	113 (24%)	
East North Central	305 (20%)	94 (20%)	
East South Central	68 (4.6%)	25 (5.3%)	
West North Central	111 (7.5%)	32 (6.8%)	
West South Central	41 (2.8%)	22 (4.7%)	
Mountain	71 (4.8%)	19 (4.0%)	
Pacific	213 (14%)	52 (11%)	
Number of missing	0	1	
Distance between zip code and treating facility (miles)	8 (4, 18)	6 (3, 16)	<0.001
Number of missing	9	1	
AJCC clinical T stage at diagnosis			0.070
T2	1232 (83%)	394 (83%)	
T3	143 (9.6%)	55 (12%)	
T4a	113 (7.6%)	23 (4.9%)	

^a^ There are 38 Hispanic patients, among which all are White, 32 are male and 6 are female.

### 3.2 Survival analysis of TMT cohort

#### 3.2.1 Overall survival

**FIGURE 2:**
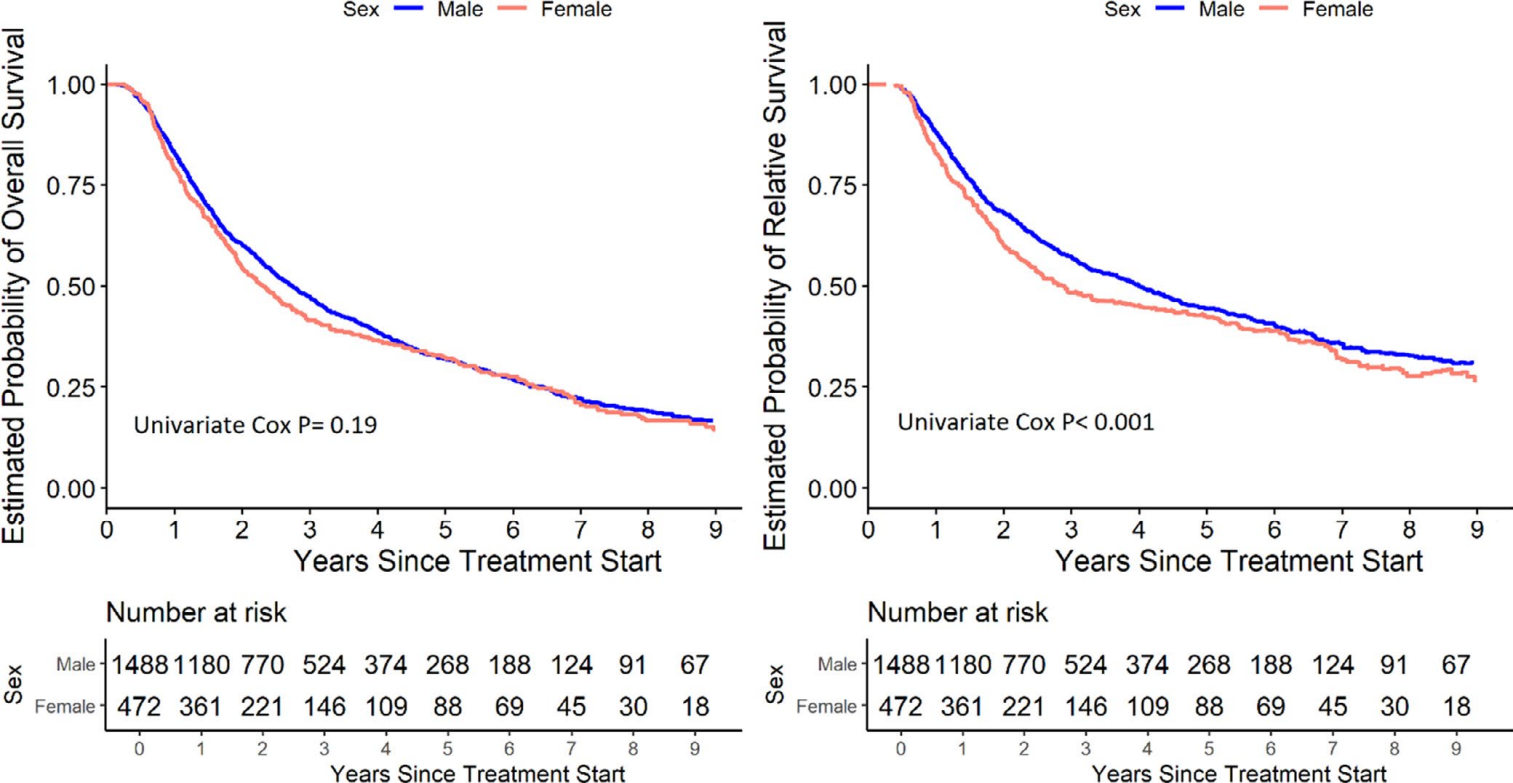
Males versus females in overall survival (A) and relative survival (B)

Kaplan–Meier estimates show no difference in long‐term OS between men and women (Figure 2A and Table S1), with an overall *p* value of 0.19, based on the Cox model with only sex as the independent variable in the model. Since inspection of these survival curves and hazard curves (data not shown) indicated that the difference between males and females was not constant over time, the 1‐year conditional survival probabilities were examined. In the first year following treatment, women had worse conditional OS compared to men (Table 2), yet not statistically significant with *p* = 0.093. The difference in OS between males and females fluctuated over time, with all other yearly conditional *p* values greater than 0.05. During Years 3 to 6, females experienced a slightly better conditional survival compared to men. Thus, the overall *p* value comparing the two curves over the full 9 years was not statistically significant; the unconditional OS at 9 years was 0.17 (95% CI [0.14, 0.20]) and 0.14 (95% CI [0.11, 0.20]) for males and females, respectively.

**TABLE 2:** Conditional survival: probability of surviving one more year given that a patient has already survived 0, 1, 2, 3, 4, 5, 6, 7, and 8 years since start of treatment

Time interval	*N* at risk at start of interval		Conditional overall survival probability (95% CI)			Relative conditional survival probability (95% CI) ^a^		
	Male	Female	Male	Female	*p*	Male	Female	*p*
[0, 1]	1488	472	0.83 [0.81, 0.85]	0.79 [0.76, 0.83]	0.093	0.88 [0.86, 0.9]	0.83 [0.79, 0.87]	0.030
[1, 2]	1180	361	0.73 [0.7, 0.75]	0.68 [0.64, 0.74]	0.16	0.77 [0.74, 0.8]	0.72 [0.67, 0.77]	0.11
[2, 3]	770	221	0.78 [0.75, 0.82]	0.77 [0.71, 0.83]	0.60	0.83 [0.8, 0.86]	0.8 [0.74, 0.86]	0.41
[3, 4]	524	146	0.82 [0.78, 0.85]	0.88 [0.83, 0.94]	0.050	0.86 [0.83, 0.9]	0.92 [0.86, 0.98]	0.094
[4, 5]	374	109	0.83 [0.79, 0.87]	0.89 [0.83, 0.95]	0.12	0.87 [0.83, 0.91]	0.92 [0.86, 0.99]	0.17
[5, 6]	268	88	0.85 [0.81, 0.9]	0.85 [0.78, 0.93]	0.99	0.89 [0.84, 0.94]	0.89 [0.81, 0.97]	0.94
[6, 7]	188	69	0.82 [0.76, 0.88]	0.75 [0.65, 0.86]	0.32	0.86 [0.8, 0.92]	0.78 [0.68, 0.9]	0.30
[7, 8]	124	45	0.85 [0.79, 0.92]	0.81 [0.7, 0.94]	0.53	0.89 [0.82, 0.96]	0.84 [0.73, 0.97]	0.55
[8, 9]	91	30	0.88 [0.82, 0.95]	0.87 [0.75, 1]	0.87	0.92 [0.85, 0.99]	0.91 [0.78, 1.06]	0.94

^a^ Calculated as conditional overall survival probability divided by expected conditional survival probability. The expected conditional survival probability is the expected survival probability of the general population matched by year, age, race, and sex conditioned on surviving through the time before each time interval. *p*‐values are based on the logrank test for overall survival and using the logrank‐like test for relative survival.

In multivariable analysis, after adjusting for the covariates listed in Table 3, women were only 1% more likely to die compared to men in the OS analysis (HR 1.01, 95% CI [0.88, 1.15], *p* = 0.94, in Table 3). Significant risk factors for OS identified by the standard multivariable Cox regression model included advanced age at diagnosis, rural residence, higher comorbidities, Medicaid or Medicare coverage or uninsured, and T3 stage or higher (Table 3).

**TABLE 3:** Cox proportional hazards regression on overall survival and relative survival from start of treatment with up to 9 years of follow‐up

	Overall survival		Relative survival	
	HR (95% CI)	*p*	HR (95% CI)	*p*
Female sex	1.01 (0.88, 1.15)	0.94	1.43 (1.25, 1.64)	<0.001
Age at diagnosis	1.03 (1.02, 1.04)	<0.001	0.94 (0.93, 0.94)	<0.001
Non‐White race	1.12 (0.9, 1.38)	0.31	0.91 (0.74, 1.13)	0.40
Median household income				
Less than $38,000				
$38,000–$47,999	0.98 (0.82, 1.18)	0.87	0.99 (0.82, 1.19)	0.94
$48,000–$62,999	0.92 (0.77, 1.11)	0.40	0.93 (0.77, 1.11)	0.42
$63,000+	0.88 (0.73, 1.06)	0.17	0.89 (0.74, 1.07)	0.20
Urban/rural residence				
Metro				
Urban	1.03 (0.87, 1.21)	0.77	1.04 (0.88, 1.22)	0.69
Rural	1.81 (1.2, 2.74)	0.005	1.84 (1.22, 2.78)	0.004
Charlson/Deyo score				
No comorbid conditions recorded				
1	1.23 (1.08, 1.41)	0.002	1.24 (1.08, 1.41)	0.002
Greater than or equal to 2	1.71 (1.43, 2.03)	<0.001	1.71 (1.44, 2.04)	<0.001
Type of insurance				
Private insurance/managed care/ other government				
Not insured/ Medicaid	1.5 (1.05, 2.14)	0.024	1.41 (0.99, 2.01)	0.056
Medicare	1.22 (1.03, 1.44)	0.019	1.24 (1.05, 1.46)	0.012
Treatment facility type				
Community cancer program				
Comprehensive community cancer program	0.92 (0.77, 1.11)	0.39	0.92 (0.77, 1.11)	0.39
Academic/research program	0.94 (0.77, 1.15)	0.55	0.95 (0.77, 1.16)	0.59
Integrated network cancer program	0.89 (0.71, 1.12)	0.32	0.9 (0.72, 1.13)	0.35
Treatment facility location				
New England				
Middle Atlantic	0.93 (0.72, 1.2)	0.57	0.93 (0.72, 1.2)	0.57
South Atlantic	0.95 (0.74, 1.23)	0.70	0.94 (0.73, 1.22)	0.66
East North Central	1.04 (0.81, 1.34)	0.76	1.03 (0.8, 1.33)	0.79
East South Central	0.7 (0.49, 1.02)	0.062	0.71 (0.49, 1.02)	0.063
West North Central	0.92 (0.68, 1.25)	0.59	0.92 (0.67, 1.24)	0.57
West South Central	1.23 (0.85, 1.8)	0.28	1.21 (0.83, 1.77)	0.31
Mountain	1.26 (0.89, 1.77)	0.19	1.27 (0.9, 1.78)	0.18
Pacific	0.83 (0.63, 1.1)	0.19	0.84 (0.64, 1.11)	0.22
Distance between zip code and treating facility	1 (1, 1)	0.33	1 (1, 1)	0.28
AJCC clinical T stage at diagnosis				
T2, T2a, or T2b				
T3, T3a, or T3b	1.36 (1.14, 1.64)	<0.001	1.36 (1.13, 1.63)	<0.001
T4 or T4a	1.35 (1.1, 1.67)	0.005	1.33 (1.08, 1.65)	0.007

#### 3.2.2 Relative survival

In contrast, when analyzing RS, the graphical estimates of long‐term RS (Figure 2B) indicated that the difference between males and females was slightly greater when compared to the Kaplan–Meier plots of OS (Figure 2A), with a resulting *p* < 0.001. Examining the 1‐year conditional RS probabilities, in the first year following treatment, women had worse conditional RS compared to men (Table 2), *p* = 0.030. As with conditional OS, the difference in RS between males and females decreased over time, with all other yearly conditional *p* values greater than 0.05; during Years 3 to 6, females experienced a slightly better conditional survival compared to men. However, for RS, the earlier difference in the first year was not fully compensated by the smaller differences in subsequent years (*p* value <0.001 over the full 9 years of follow‐up). The unconditional RS at 9 years was 0.31 (95% CI [0.27, 0.37]) and 0.27 (95% CI [0.20, 0.36]) for males and females, respectively.

In multivariable analysis after adjusting for the covariates listed in Table 3, women were 43% more likely to die compared to men in the RS analysis (HR 1.43, 95% CI [1.25, 1.64], *p* < 0.001, in Table 3). Significant risk factors for RS on multiplicative Cox regression included female status, younger age at diagnosis, rural residence, higher comorbidities, Medicare insurance, and T‐stage ≥T3 (Table 3).

## 4 DISCUSSION

In this large retrospective cohort study of patients with MIBC undergoing TMT, we did not observe a consistent inferior OS outcome among women. We did, however, find that women had an initial higher risk of death compared to men during the first year following treatment initiation. With over 1960 patients, this is the largest report to compare oncologic outcomes between men and women undergoing TMT.

The difference in survival between men and women within the first year following TMT may be related to understaging. Clinical staging and pathologic staging in bladder cancer vary; at cystectomy, approximately 40% of patients have been shown to be clinically understaged.^18^, ^19^ Understaging has been reported to be more common in women than men in some series, but not universally agreed upon.^3^, ^9^, ^20^, ^21^ Surgical upstaging was associated with decreased survival for all stages.^18^, ^19^ Understaging in women may be related to delayed diagnosis in women.^3^, ^4^, ^5^, ^6^ Perhaps what is being detected in our RS analysis is that the women who survive past 1 year are staged more accurately and are more likely to have an organ‐confined disease. Similarly, the impact of advanced disease may be seen earlier (e.g., stage is not significant in the conditional analyses in Years 2–5 and 5–9).

Understaging of women prior to TMT could also be related to anatomic difference in the bladder wall thickness between men and women; with a thinner bladder wall, women may be at increased risk of micrometastatic disease at diagnosis.^22^

Another explanation for women having worse RS than men initially may be related to molecular subtype. Molecular subtyping of bladder cancer has shown survival differences based on subtype.^23^ Recent data have shown that female patients are more likely to develop basal/squamous subtype than males.^7^ Thus, it has been proposed that women may do worse following RC based on the more aggressive subtype.^7^ This may be the case for TMT, as well.

While molecular subtype does have a differential response to neoadjuvant chemotherapy, Efstathiou et al. demonstrated that the rate of complete response, DSS, nor OS differed at 5 years based on subtype following TMT.^24^ It is unclear from their analysis whether there was a difference in DSS within the first year.

A group from the Netherlands similarly found worse RS in women compared to men when evaluating their long‐term cancer registry data.^22^ Women had 1.5 times higher than excess risk of mortality compared to men in the first 2 years following diagnosis. This was seen in MIBC and non‐invasive bladder cancers and was seen regardless of treatment (RC vs. TMT). There was, however, no evaluation of the effect of sex on specific treatments chosen (RC or TMT).^22^

A prior institutional review of patients treated at Erlangen University Hospital compared long‐term outcomes between 286 men and 105 women treated with either RT alone or TMT between 1982 and 2007 with 5‐year follow‐up.^25^ They found that females did not present with more advanced disease than men but did find that female sex was an independent prognostic factor for worse cancer‐specific survival and OS. However, male patients were more likely to receive CRT than females (71.2% vs. 65.7%), and men received a slightly higher dose of radiation; both factors likely contributed to worse outcomes for women. Of note, the mean overall RT dose for both men and women in this study was lower than what would be expected for TMT and so it is unclear if the patients included on this study were all being treated with curative intent or if they were having higher numbers of early salvage cystectomies, which is also associated with decreased OS.

The NCDB does not capture DSS, which is a limitation to this study. In order to overcome that limitation, we used OS as the oncologic outcome of interest by comparing the observed OS to the expected survival of the general population and calculating the RS. In addition, interpretation of the patterns of differences between males and females was complicated by the fact that the sex variable violated the assumption of proportional hazards; the effect of this is that in the standard Cox multivariate proportional hazards model, early differences could be obscured by later patterns—that is, attenuating the differences between males and females. To address this possibility, conditional 1‐year probabilities for survival were examined.

Additional limitations to the current study include those inherent in any registry study: inaccurate or incomplete classification of tumor characteristics and/or causes of death, lack of centralized review of pathology affecting grade and histologic subtype, inability to determine whether the pre‐TMT TURBT was a maximal resection, absence of more specific data on chemotherapeutic regimens, inability to know what imaging modalities or examinations were used to define the clinical stage, and subsequent treatments all of which may lead to unmeasured confounding and bias our findings towards the null hypothesis.

## 5 CONCLUSION

In this analysis of the NCDB, we found that women had a higher initial risk of death compared to men in the first year following TMT. There were, however, no long‐term differences in OS between sexes. TMT remains an important treatment option in both men and women seeking bladder preservation and not does not likely contribute to gender disparities in disease progression or mortality.

## CONFLICT OF INTEREST

All authors have completed a conflict of interest disclosure and have no conflicts of interest that pertain to this study.

## Supporting information

Table S1

## Data Availability

All data generated and analyzed during this study are included in this published article (and its supporting information files). Research data are stored in an institutional repository and will be shared upon request to the corresponding author. This project was reviewed and approved by the USC IRB and is registered as HS‐19–00854.
