# Supplementary material for: Disparities in male versus female oncologic outcomes following bladder preservation: A population‐based cohort study
Source: Cancer Med. 2021 Mar 28;10(9):3004–12. doi: 10.1002/cam4.3835 (PMC8085939; doi:10.1002/cam4.3835)
Supplement: Supplementary file 1 — Table S1 [file CAM4-10-3004-s001.docx]

**Supplemental Table S1.** Males vs. Females in Overall Survival and Relative Survival based on Figure 2.

| **Years**  **since TX** | **Overall Survival Probability (95%CI) – Figure 2A** | | **Relative Survival Probability (95%CI) – Figure 2B** | |
| --- | --- | --- | --- | --- |
|  | **Male** | **Female** | **Male** | **Female** |
| **1** | **0.83 (0.81, 0.85)** | **0.79 (0.75, 0.83)** | **0.88 (0.86, 0.9)** | **0.83 (0.79, 0.87)** |
| **2** | **0.6 (0.58, 0.63)** | **0.54 (0.5, 0.59)** | **0.68 (0.65, 0.71)** | **0.6 (0.55, 0.65)** |
| **3** | **0.47 (0.45, 0.5)** | **0.42 (0.37, 0.47)** | **0.57 (0.54, 0.6)** | **0.48 (0.43, 0.54)** |
| **4** | **0.39 (0.36, 0.41)** | **0.37 (0.32, 0.42)** | **0.5 (0.47, 0.54)** | **0.45 (0.4, 0.51)** |
| **5** | **0.32 (0.29, 0.35)** | **0.32 (0.28, 0.38)** | **0.44 (0.41, 0.48)** | **0.43 (0.37, 0.49)** |
| **6** | **0.27 (0.24, 0.3)** | **0.28 (0.23, 0.33)** | **0.4 (0.37, 0.45)** | **0.39 (0.33, 0.46)** |
| **7** | **0.22 (0.2, 0.25)** | **0.21 (0.16, 0.26)** | **0.36 (0.32, 0.4)** | **0.32 (0.25, 0.4)** |
| **8** | **0.19 (0.16, 0.22)** | **0.17 (0.13, 0.22)** | **0.33 (0.28, 0.38)** | **0.28 (0.21, 0.36)** |
| **9** | **0.17 (0.14, 0.2)** | **0.14 (0.11, 0.2)** | **0.31 (0.27, 0.37)** | **0.27 (0.2, 0.36)** |
